# Molecular Imaging with MRI: Potential Application in Pancreatic Cancer

**DOI:** 10.1155/2015/624074

**Published:** 2015-10-22

**Authors:** Chen Chen, Chang Qiang Wu, Tian Wu Chen, Meng Yue Tang, Xiao Ming Zhang

**Affiliations:** Sichuan Key Laboratory of Medical Imaging, Department of Radiology, Affiliated Hospital of North Sichuan Medical College, Wenhua Road 63, Nanchong, Sichuan 637000, China

## Abstract

Despite the variety of approaches that have been improved to achieve a good understanding of pancreatic cancer (PC), the prognosis of PC remains poor, and the survival rates are dismal. The lack of early detection and effective interventions is the main reason. Therefore, considerable ongoing efforts aimed at identifying early PC are currently being pursued using a variety of methods. In recent years, the development of molecular imaging has made the specific targeting of PC in the early stage possible. Molecular imaging seeks to directly visualize, characterize, and measure biological processes at the molecular and cellular levels. Among different imaging technologies, the magnetic resonance (MR) molecular imaging has potential in this regard because it facilitates noninvasive, target-specific imaging of PC. This topic is reviewed in terms of the contrast agents for MR molecular imaging, the biomarkers related to PC, targeted molecular probes for MRI, and the application of MRI in the diagnosis of PC.

## 1. Introduction

Pancreatic cancer (PC) is a serious threat to human health, due to malignant tumors with concealed onset, rapid development, and poor prognosis. PC is the fourth leading cause of death among all cancers in the USA, with a dismal 5-year survival rate of less than 5% [1]. These dismal outcomes can be attributed to the lack of early diagnoses and the inability to detect precancerous lesions [2]. Therefore, the detection and diagnosis of PC in the early stage are extremely urgent. At present, the methods used to diagnosis PC include tumor marker detection and imaging diagnosis. The traditional tumor markers that have been used for the early diagnosis of PC have high sensitivity in clinical use, but the specificities are not high, and these markers are thus prone to false positives [3]. Computed tomography (CT) and magnetic resonance imaging (MRI) have been used to diagnose and stage the majority of PCs with tumor detection limits of 5–8 mm, when the earliest precursor lesions are in the microscopic range [4, 5]. Nevertheless, the development of molecular imaging technology enabled the effective resolution of this difficulty. Molecular imaging is a medical imaging technique that combines molecular biology, chemistry, material science, radiation medicine, and computer science and has created a profile for the diagnosis and treatment of this disease that exhibits wide application prospects from the bench to the clinic [6–9]. In contrast with traditional imaging techniques that are primarily based on gross anatomy structures, molecular imaging can identify pathological changes at the molecular and cellular level, determine the qualitative properties of the diseases, enable objective monitoring of the efficacy of treatment, and predict disease development. Molecular imaging research primarily includes two aspects, the first of which is the choice of imaging equipment. Molecular MR imaging has become a novel technique for assessing specific cellular or subcellular events and is becoming one of the core integrative technologies in biomedicine because many of the parameters that are used to produce contrast, such as the spin-lattice relaxation (*T*_1_) and spin-spin relaxation (*T*_2_) times, are dependent on the local chemical structure of the molecules being imaged [10]. In most situations, near-infrared optical fluorescence (NIRF) imaging is combined with MRI, which enables the direct visualization of the enriched area of the tumor in the visible range [11]. The second aspect is the preparation of the targeting probes of the equipment. Molecules or cells with reporter genes or imaging agent are introduced into the body and used to observe changes at the molecular and cellular levels based on the identifying agent [12, 13]. The present study reviews the contrast agents for MR molecular imaging, related biomarkers for PC, targeted molecular probes for MRI, and their applications in the diagnosis of PC.

## 2. Contrast Agents for MR Molecular Imaging

At present, there are two main types of MR contrast agent. The first are Gd3 + paramagnetic compounds, which can produce *T*_1_-weighted imaging (*T*_1_WI) positive contrasts. Currently, small gadolinium-containing contrast agents, such as gadopentetate dimeglumine (Gd-DTPA, Magnevist), are among the most widely used in MR molecular imaging. Because Gd-DTPA has a low molecular weight, after injection into the body, it can travel through the capillary into the intercellular space and be distributed nonspecifically. Because Gd-DTPA cannot pass through the blood-brain barrier, the contrast agent concentration achieves equilibrium rapidly in normal tissues and lesion areas [14]. The known adverse reactions to the use of DTPA include nausea, urticaria, and taste disorder. Among these reactions, the most serious is nephrogenic systemic fibrosis (NSF). This type of adverse reaction results when macrophages engulf free gadolinium and subsequently release cytokines that promote fibrosis or when gadolinium complexes are engulfed by peripheral blood mononuclear cells, which then release proinflammatory cytokines that eventually lead to tissue fibrosis [15, 16].

Another type of contrast agent is superparamagnetic iron oxide (SPIO) nanoparticles, such as Fe_2_O_3_ and Fe_3_O_4_, which mainly produce *T*_2_-weighted imaging (*T*_2_WI) with negative contrast. Compared with the Gd-DTPA, SPIO elicits lower contrast medium toxicity because the SPIO nanoparticles that are released from dying cells can be degraded in the normal iron recycling pathways [17]. Simultaneously, SPIO improves the biocompatibility and the blood retention time and increases the contrast intensity [18]. To our knowledge, the value of SPIO for targeted imaging lies in the fact that the SPIO surface can be packaged and subsequently combined with appropriate targeting ligands. In recent years, some scholars [19–23] have studied the design scheme and biological characteristics of the molecular imaging application of SPIO and believe that to ensure that SPIO has hydrophobic and certain toxic properties and is uniformly distributed in the ferrofluid the selection of the surface package material is critical. The material used for the surface coating of the magnetic particles not only must be nontoxic and biocompatible but also must allow the targetable delivery with particle localization within a specific area. In recent studies, inorganic silicon materials [24–27], polyacrylic acid [28, 29], dextran [30, 31], dopamine [32], deferoxamine [33–35], and other organic polymers have been used for the surface packaging of SPIO.

Manganese is a nonlanthanide paramagnetic metal that possesses good relaxation enhancement effects, due to the five unpaired electrons of bivalent manganese. Because manganese not only plays essential roles in cell biology but also is minimally toxic in vivo, large doses can be used in MRI. Manganese-based contrast agents include a variety of forms, such as small organic chelates [36], macromolecule chelates [37], and oxide nanoparticles.

In general, after the contrast agent is packaged, appropriate targeting ligands need to be selected based on the research target or a specific type of cell. Pancreatic cancer is well known to express a variety of biomarkers; therefore, increasing the sensitivity and specificity of markers and their corresponding ligands is the main goal of research in pancreatic cancer-targeted imaging.

## 3. Related Biomarkers for PC

The increasing study of pancreatic cancer has established that pancreatic cancer is a highly heterogeneous disease involving extremely complex tumor microenvironments that express a variety of antigens and receptors within the tumor cells and surrounding stroma. These related proteins and highly expressed genes in malignant tumors are the design foundation of functionally targeted nanoparticles.

### 3.1. Related Serum Biomarkers for PC

Among the numerous biomarkers that have been tested for PC detection, serum CA19-9 is the most commonly used. CA19-9 is a type of carbohydrate antigen that exists at the cell surface and is associated with a variety of digestive tract tumors. However, CA19-9 lacks the sensitivity needed to detect early-stage PC [38] and to monitor responses to therapy, because of its poor sensitivity (41%–86%) and specificity (33%–100%) [39]. Furthermore, CA19-9 can also arise in some benign lesions, such as bile duct inflammation, chronic pancreatitis, and other gastrointestinal cancers [40], and tends to arise only after tumor metastasis [3]. Kim et al. [41] used CA19-9 to screen for PC in 70,940 asymptomatic patients. Among the 1,063 patients with elevated levels, only 4 had pancreas cancer, and only 2 had resectable disease. Therefore, in some special conditions, the accuracy and specificity of the use of CA19-9 as a target are also controversial views.

Muc-1 is a transmembrane mucin glycoprotein and is another biomarker that is associated with the most invasive forms of PC [42]. Muc-1 levels are elevated in the majority of patients with PC, and Muc-1 plays a key role that affects oncogenesis and the motility, metastasis, metabolism, and growth of cancer cells [43, 44]. Gold et al. [45] proved that Muc-1 is overexpressed in PC both in the cytoplasm and in the cell membrane, compared with most chronic pancreatitis tissues and normal pancreatic tissues in which Muc-1 is only expressed in the cell membrane with no cytoplasm expression. Thus, there is a direct relationship between high invasiveness and poor PC prognosis [46, 47]. The PAM4 antibody against Muc-1 is more specific for pancreatic cancer than antibodies to the other Muc-1 antigens that are observed in other tumors. In a recent study [48], the authors found that the PAM4-reactive Muc-1 epitope was not detected in the normal pancreas but was expressed in 87% (48 of 55) of invasive pancreatic adenocarcinomas. Additionally, Muc-1 acts as a master regulator of the metabolic program that can also help tumor cells survive and proliferate in hypoxic environments [43]. Many studies [42, 49–51] have demonstrated that Muc-1 can be used as an ideal target in the diagnosis and treatment of pancreatic cancer.

Survivin is a newly identified member of the apoptosis inhibitory protein family and has highly specific tissue distribution and powerful antiapoptotic function. Ren et al. [52] analyzed the serum levels of survivin in patients with pancreatic ductal adenocarcinoma (PDAC) (*n* = 80) and age-matched healthy volunteers (*n* = 80) and found that the serum survivin concentrations were significantly elevated in the sera of PDAC patients compared with healthy sera (*p* = 0). Dong et al. [53] performed a similar study and reached similar conclusions. Thus, the expression of the survivin protein is closely related to the biological characteristics of pancreatic tissue.

Currently, the receptors known to be related to PC mainly consist of chemokine epidermal growth factor receptor 4 (CXCR-4), vascular endothelial growth factor (VEGF), epidermal growth factor receptor (EGFR), and urokinase plasminogen activator receptor (UPAR).

CXCR-4 is the specific receptor of chemotactic factor CXCL12. Many lines of evidence indicate that the CXCL12/CXCR-4 biological axis plays an important role in the proliferation, invasion, and metastasis of PC [54–56] and is a suitable target for therapy and imaging [55, 57, 58].

EGFR is a member of the HER family, which is particularly highly expressed in malignant tumors with epithelial tissue sources. In pancreatic cancer tissue, the expressions of the differentiation of different statuses are also different.

Currently, VEGF is the most potent and specific angiogenic factor that directly affects vascular endothelial cells. In most situations, VEGFR is expressed in new vascular endothelial cells within the tumor. PC is associated with a lack of blood supply. Nonetheless, VEGFR has been found not only in blood vessels but also in blood vessel cells [59]. Furthermore, Karayiannakis et al. [60] reported that PC patients have significantly higher VEGF levels than healthy controls and that serum VEGF levels are significantly associated with disease stage and the presence of both lymph node and distant metastases.

UPAR is a versatile signaling orchestrator of cellular differentiation, proliferation, and migration [61]. Researchers recently discovered that UPAR is expressed in PC tissues at rates not less than 86%, whereas UPAR is not found in pancreatic tissues obtained from healthy subjects or patients with chronic pancreatitis [62–64]. A recent study revealed that, among the 27 genes that are commonly used in PC tissues, the level of UPAR exhibited the highest accuracy in the differential diagnosis of pancreatic ductal carcinoma and chronic pancreatitis [64]. Additionally, desmoplasia and hypovascularity are the pathological hallmarks of pancreatic tumors [65]. One study found that UPAR is highly expressed in tumor and stroma cells [66, 67]. Thus, UPAR may have very broad application prospects in PC molecular imaging research.

Additionally, some protein markers have newly been discovered. CEACAM-1 [68, 69], CEACAM-6 [70–72], CD133 [73–75], S100A4 [76–80], and midkine [81] have been shown to be biomarkers that are also expressed in PC and are significantly associated with invasion and metastasis in PC and PC prognosis. Therefore, these markers also have the potential to become the imaging and therapy targets for PC.

### 3.2. Related miRNA for PC

Currently, more than 20 miRNAs have been proven to be associated with PC [82]. miRNA-21 has been considered to be the miRNA most closely related to cell proliferation, metastatic ability, and poor overall survival [83–86]. Moreover, miRNA-21 has been demonstrated to be significantly overexpressed in both PC cell lines and tissues relative to normal pancreatic tissue [87]. Additionally, some other miRNAs (130b [88, 89], 196a [90, 91], 92a [92, 93], 198 [94], 221 [95, 96], 23b [97], and 29a [98]) have also been shown to have important roles in PC. In a recent study, Nagano et al. [99] established 7 miRNA-based biomarker models (miR-20a, miR-21, miR-24, miR-25, miR-99a, miR-185, and miR-191) for PDAC diagnosis and found that these biomarkers exhibited high sensitivity and specificity in the discrimination of PC and chronic pancreatitis patients (AUC = 0.993). Therefore, the identification of the miRNAs suggests that they can also be used as potential tools for the screening of early-stage PC.

### 3.3. Genes Related to PC

Currently, many differentially expressed genes related to signal transduction are known to play roles in the development of PC that include the stimulation of protooncogenes, such as K-ras [100, 101], HER-2/neu [102, 103], and BRCA [104, 105], and the inactivation of tumor suppressor genes, such as SMAD4 [106], APC [107], P53 [108, 109], and CDKN2A [110, 111]. The associated genes that have been identified as being involved in these processes have potential as imaging markers for PC.

Although, at present, a wide variety of tumor markers have been associated with PC, these markers cannot fully meet the requirements of imaging targets of PC, primarily because the sensitivity, specificity, and expression quantities are not homogenous. Nonetheless, additional exploration and in-depth study are needed to select the appropriate molecular imaging targets for PC.

## 4. MR Target Molecular Imaging for PC

**Figure 1:**
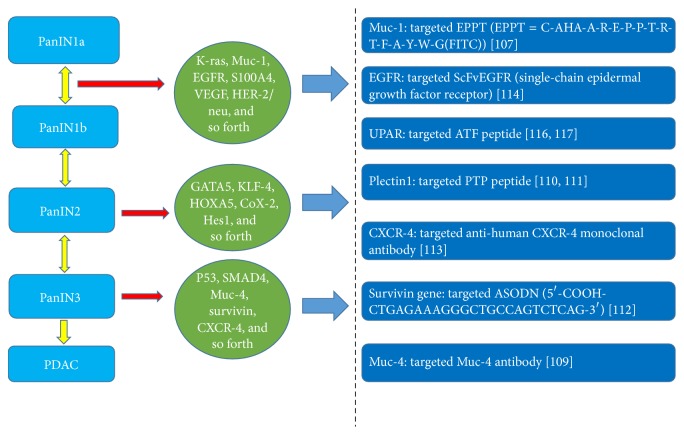
As PC progresses from PanINs to PDAC, each stage is well characterized by multiple molecular alternations. However, the identification of specific lesions using unique molecular markers as early as possible through molecular imaging will lead to the early detection of this deadly disease. The right illustrates the target materials that correspond to different PC biomarkers.

**Figure 2:**
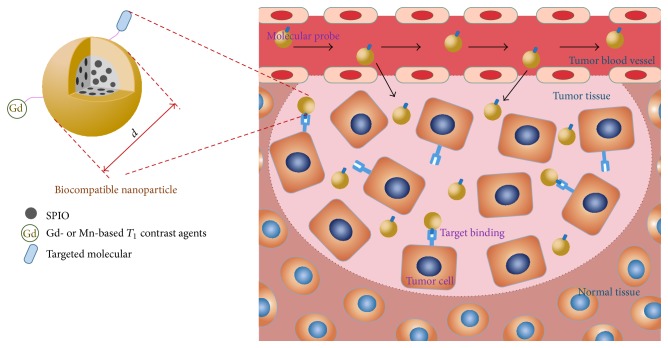
Targeted molecular MRI probes for the early diagnosis of pancreatic cancer (PC). Superparamagnetic iron oxide (SPIO) or paramagnetic metal complexes (Gd- or Mn-based *T*_1_ contrast agents) are loaded in biocompatible nanoparticles that molecularly target the surface and are suitable at the nanoscale levels (*d* = 10~100 nm). The nanocomposites can reach the tumor tissue through tumor blood vessel clearance and target and bind tumor cells to alter the signal intensity of the tumor tissue on MRI.

In the MR molecular imaging of PC, the key step is the preparation of the appropriate targeted molecular probes for MRI. First, an MRI molecular probe must have high specificity which can distinguish the PC from the surrounding tissues. Second, an MRI molecular probe must exhibit a high sensitivity for identifying the subtle changes in the early stage of PC (Figure 1). Additionally, an MRI molecular probe must also exhibit excellent biological compatibility that can overcome a variety of physiological barriers in the body and minimize side effects to the greatest possible extent (Figure 2). Thus, the selection of imaging probes with the above-mentioned characteristics is the primary focus and most difficult aspect of the research field of MR target molecular imaging.

### 4.1. Molecular Imaging Probes Targeting the Muc Protein

In 2006, Medarova et al. [112] prepared a dual-modality imaging probe that specifically targeted the underglycosylated mucin-1 tumor-specific antigen (uMuc-1). This probe is comprised of cross-linked superparamagnetic iron oxide (CLIO) nanoparticles and peptides (EPPT) that specifically recognize uMuc-1, which is attached to the nanoparticles' dextran coats. After the injection of CLIO-EPPT in orthotopic pancreatic cancer mice, the average *T*_2_ relaxation rate of the PC tissues significantly decreased, whereas that of the muscle tissues was unaffected. These authors concluded that the CLIO-EPPT contrast agent could be targeted to PC tissues and result in dramatic signal changes, and related iron oxides are already in clinical use [113]. In the mucin family, another membrane-bound mucin gene, Muc-4, is expressed at a high level in PC and has not yet been found in chronic pancreatitis or normal pancreatic tissues [114]. Wu et al. [115] developed the Muc-4-targeting SPIO contrast agent MnMEIO-silane-NH_2_-(Muc-4)-mPEG NPs, which exhibited better negative contrast enhancement and did not interfere with the MR images. In animal experiments, a *T*_2_-weighted MR study revealed that this novel contrast agent could specifically and effectively target mucin-4-expressing pancreatic tumors in nude mice. In the *T*_2_-weighted imaging study by these authors, they demonstrated that the intensity of negative contrast enhancement was marked in the HPAC tumor cells in which Muc-4 was expressed at a high level compared with the Panc-1 tumor cells, which exhibited significantly lower negative contrast enhancement due to lower Muc-4 expression.

### 4.2. The Molecular Imaging Probe Targeting Plectin1

Plectin1 exhibits distinct cytoplasmic and nuclear localization in normal fibroblasts but exhibits aberrant expression on the cell membrane in pancreatic ductal adenocarcinoma (PDAC). In one study [116], Plectin1 targeted peptides (PTP) were conjugated to the surface of magnetofluorescent nanoparticles, and the results revealed that the targeted imaging agent PTP-NP permitted imaging of PDAC against the background of normal and ductal metaplasia of the pancreas. In intravital MRI, these nanoparticles enabled the detection of small PDACs and precursor lesions in engineered mouse models that exhibited a reduction in MR signal in the PDAC regions. Furthermore, the results were confirmed by histological analysis, and fluorescence microscopy indicated that the loss of signal associated with PTP-NP uptake occurred primarily in the regions of PDAC and not in the normal regions or regions of ductal metaplasia. In another study, Wang et al. [117] developed the novel targeted imaging contrast agent dyeBSA·SPIONs-mAb. Panc-1 cell MR scanning was performed following incubation with Plec-1-targeted dyeBSA·SPIONs-mAb. This study demonstrated that a significant reduction in *T*_2_ reduction occurred compared with the nontargeted dyeBSA·SPIONs group at the same concentration. These studies that reported the development of a specific imaging probe and the discovery of Plectin1 as a novel biomarker may have clinical utility in the diagnosis of PDAC in humans.

### 4.3. The Molecular Imaging Probe Targeting the Survivin Gene

More recently, the survivin gene, which is a potential marker of PC, has been regarded as a targeting gene, and chitosan-coated magnetic iron oxide particles (MNPs) have been regarded as imaging probes for the detection of PC [19]. Chitosan-coated MNPs (cs@MNPs) and antisense oligodeoxynucleotides of the survivin gene were conjugated to MNPs to produce Sur-MNPs. The magnetic resonance signal intensities of the pancreatic cells labeled with cs@MNPs, MNPs, and Sur-MNPs were compared on *T*_2_-weighted images. Ultimately, these authors found that the Sur-MNPs exhibited a proper size, high stability, not cytotoxicity, and good dispersion compared with the others. More importantly, the Sur-MNPs did not accumulate in healthy lung fibroblast cells (in the control group) but were taken up by BxPC-3 cells (expressing the survivin gene) and exhibited low signal due to the *T*_2_-weighted effect. Thus, our research not only demonstrated that the survivin gene of PC was detectable by Sur-MNPs but also indicated that Sur-MNPs may become good negative molecular contrast agents in the diagnosis of PC. Further studies evaluating the selective uptake of Sur-MNPs in PC xenografts in vivo are extremely urgent.

### 4.4. The Molecular Imaging Probe Targeting CXCR-4

In 2012, He et al. [118] reported a study of the anti-CXCR-4 monoclonal antibody conjugated to ultrasmall superparamagnetic iron oxide nanoparticles (CXCR-4-USPIO) in an application of MR molecular imaging of PC cells. The results indicated that the CXCR-4-USPIO group not only exhibited lower *T*_2_ values compared with the BSA-USPIO group but also exhibited a high affinity with the PC cells according to the MR imaging. Additionally, the *T*_2_ enhancement ratio and Δ*R* ^2^ values of the CXCR-4-USPIO nanoparticles were useful for semiquantitatively assessing the cellular CXCR-4 expression levels. However, the defect of this study was the lack of an orthotopic human pancreatic cancer xenograft animal model to evaluate the in vivo contrast enhancement imaging efficacy of the CXCR-4-USPIO probe, and this issue will be our research direction in the future.

### 4.5. The Molecular Imaging Probe Targeting EGFR

EGFR is a member of the HER family. In a recent study [119], single-chain epidermal growth factor receptor antibody- (ScFvEGFR-) conjugated quantum dots (QDs) or magnetic iron oxide (IO) nanoparticles were used for tumor target imaging in vivo. This study revealed that the uptake of targeted IO nanoparticles selectively occurred in PC cells, a finding confirmed by positive Prussian blue staining results, whereas the normal pancreatic ductal epithelial cells and other normal cell types were negative for this staining. In an in vivo experiment, after the EGFR-targeted MRI of human pancreatic cancer orthotopically implanted into the pancreas of nude mice, it was shown that the ScFvEGFR-IO nanoparticles selectively accumulated within the pancreatic tumors in *T*_2_-weighted fast spin echo imaging, as evidenced by a decrease in the MRI signal in the area of the tumor. In a similar study [120], Yang et al. conjugated ScFvEGFR fragments with magnetic iron oxide (IO) NPs to obtain ScFvEGFR-IOs and investigated their binding and internalization by EGFR-expressing cancer cells. Using the MRI technique, these investigators demonstrated that ScFvEGFR-IO specifically bound to and was internalized by EGFR-expressing cancer cells. Additionally, the use of ScFvEGFR-IO as a molecular imaging agent was demonstrated with MRI in an orthotropic human pancreatic cancer mouse xenografted model.

### 4.6. The Molecular Imaging Probe Targeting UPAR

More recently, attempts have been made to identify potential imaging probes for the active targeting of the pancreatic stroma. UPAR is a biomarker of PC that is highly expressed in tumor and stroma cells, and the active retention of these nanoparticles is increased in many target cells in tumor masses. Yang et al. [121] designed a dual mode of molecularly targeted agents that involved Fe_2_O_3_ nanoparticles conjugated with near-infrared dyes and uPA at the same times. The MR imaging results indicate that the systemic delivery of the UPAR-targeted nanoparticles led to their selective accumulation in the orthotopically xenografted human PC tumors in nude mice, and MRI signal reduction was detected in the UPAR-expressing cells. The probe binds to and is subsequently internalized by UPAR-expressing tumor cells and tumor-associated stroma cells. In 2013, Lee et al. [122] engineered urokinase plasminogen activator receptor- (UPAR-) targeted magnetic iron oxide nanoparticles (IONPs) that carry the chemotherapy drug gemcitabine (Gem) for targeted delivery into UPAR-expressing tumor and stroma cells in MRI. The results revealed that UPAR can act not only as the imaging probe but also as the therapy carrier for PC.

### 4.7. The Molecular Imaging Probe Targeting Some Antibodies and Receptors

Pirollo et al. [123] designed a tumor-targeting, liposomal nanodelivery platform to improve the early detection of tumors with MRI. These authors used Gd-DTPA in an anti-transferring receptor single-chain antibody (TfRscFv) liposomal complex and injected this complex into an animal model of PC. The results revealed that this compound significantly increased the signal of the lesion area and improved the contrast between the lesion and normal tissues, which aided the localization and qualitative diagnosis of PC. In 2006, Montet et al. [124] designed a nanoparticle-conjugate targeted to the bombesin (BN) receptors present on the normal acinar cells of the pancreas. In this study, the authors found that the BN-CLIO nanoparticles decreased the *T*_2_ signal of the normal pancreas and enhanced the ability to visualize the tumor on MRI in a model of pancreatic cancer. Additionally, some contrast agents, such as the GO-IONP, exhibited powerful abilities for the dual-modality mapping of the regional lymphatic system by MRI [125].

## 5. Summary

MR molecular imaging appears to be a promising imaging modality for the early detection of PC. This imaging modality also facilitates the study of the pathological changes associated with PC at the molecular and cellular levels. Regarding this topic, we summarize the applications of MR molecular imaging in the diagnosis of PC. As a noninvasive, target-specific imaging modality, MR molecular imaging can not only improve the early detection of PC but also be modified for the targeted selectivity of tumor cells to increase imaging resolution. At present, many studies have conducted in vivo experiments and provided evidence of the feasibility of these targeted contrast agents. However, there are still some studies that have not conducted in vivo experiments. Therefore, this issue is worthy of extensive research because these issues have great significance for targeted molecular imaging and therapy of PC. Currently, the research related to the MR molecular imaging of PC is still in its infancy phase; however, in view of the existing achievement, we believe that these studies will have a far-reaching influence on the diagnosis and treatment of PC.
